# RERE deficiency leads to decreased expression of GATA4 and the development of ventricular septal defects

**DOI:** 10.1242/dmm.031534

**Published:** 2018-08-28

**Authors:** Bum Jun Kim, Hitisha P. Zaveri, Valerie K. Jordan, Andres Hernandez-Garcia, Daron J. Jacob, Diana L. Zamora, Wei Yu, Robert J. Schwartz, Daryl A. Scott

**Affiliations:** 1 Department of Molecular and Human Genetics, Baylor College of Medicine, Houston, TX 77030, USA; 2 Department of Molecular Physiology and Biophysics, Baylor College of Medicine, Houston, TX 77030, USA; 3 Department of Biology and Biochemistry, University of Houston, Houston, TX 77004, USA

**Keywords:** RERE, GATA4, Atrioventricular canal, Atrioventricular cushion, Endothelial-to-mesenchymal transition, Ventricular septal defects

## Abstract

Deletions of chromosome 1p36 are associated with a high incidence of congenital heart defects (CHDs). The arginine-glutamic acid dipeptide repeats gene (*RERE*) is located in a critical region for CHD on chromosome 1p36 and encodes a cardiac-expressed nuclear receptor co-regulator. Mutations affecting *RERE* cause atrial and ventricular septal defects (VSDs) in humans, and RERE-deficient mice also develop VSDs. During cardiac development, mesenchymal cells destined to form part of the atrioventricular (AV) septum are generated when endocardial cells in the AV canal undergo epithelial-to-mesenchymal transition (EMT) and migrate into the space between the endocardium and the myocardium. These newly generated mesenchymal cells then proliferate to fill the developing AV endocardial cushions. Here, we demonstrate that RERE-deficient mouse embryos have reduced numbers of mesenchymal cells in their AV endocardial cushions owing to decreased levels of EMT and mesenchymal cell proliferation. In the endocardium, RERE colocalizes with GATA4, a transcription factor required for normal levels of EMT and mesenchymal cell proliferation. Using a combination of *in vivo* and *in vitro* studies, we show that *Rere* and *Gata4* interact genetically in the development of CHDs, RERE positively regulates transcription from the *Gata4* promoter and GATA4 levels are reduced in the AV canals of RERE-deficient embryos. Tissue-specific ablation of *Rere* in the endocardium leads to hypocellularity of the AV endocardial cushions, defective EMT and VSDs, but does not result in decreased GATA4 expression. We conclude that RERE functions in the AV canal to positively regulate the expression of GATA4, and that deficiency of RERE leads to the development of VSDs through its effects on EMT and mesenchymal cell proliferation. However, the cell-autonomous role of RERE in promoting EMT in the endocardium must be mediated by its effects on the expression of proteins other than GATA4.

This article has an associated First Person interview with the first author of the paper.

## Abstract (precis)

**Summary:** In the developing atrioventricular canal, RERE promotes endothelial-to-mesenchymal transition and mesenchymal cell proliferation by positively regulating *Gata4*. Tissue-specific ablation of *Rere* in the endocardium causes ventricular septal defects.

## INTRODUCTION

Deletions of chromosome 1p36 are the most common terminal deletions in humans (Heilstedt et al., 2003a). Approximately 1 in 5000 newborns carries a terminal or interstitial deletion affecting chromosome 1p36. These children are at risk for a variety of medical problems – developmental delay, intellectual disability, brain malformations, eye defects, hearing loss, congenital heart defects (CHDs), cardiomyopathy, renal anomalies, postnatal growth deficiency and dysmorphic features – which, together, constitute the 1p36 deletion syndrome (Battaglia et al., 2008; Shapira et al., 1997). Deletion of either the distal or proximal critical region on chromosome 1p36 is sufficient to cause each of these medical problems (Heilstedt et al., 2003b; Jordan et al., 2015; Kang et al., 2007).

CHD is present in ∼70% of individuals with 1p36 deletions, with 23% having ventricular septal defects (VSDs) and 28% having atrial septal defects (ASDs) (Battaglia et al., 2008). Five cardiac critical regions have been identified on chromosome 1p36, each of which is hypothesized to harbor one or more genes that play a crucial, dose-dependent role in cardiac development (Zaveri et al., 2014). The arginine-glutamic acid dipeptide repeats gene (*RERE*) is located in the proximal critical region for 1p36 deletion syndrome and in one of the five 1p36 cardiac critical regions (Jordan et al., 2015; Zaveri et al., 2014). *RERE* encodes a nuclear receptor co-regulator that is expressed in a wide variety of tissues, including the embryonic and adult heart, and has been shown to positively regulate retinoic acid signaling during development (Vilhais-Neto et al., 2010; Wang et al., 2008; Zoltewicz et al., 2004).

Recently, mutations affecting *RERE* have been shown to cause a new genetic syndrome, neurodevelopmental disorder with or without anomalies of the brain, eye or heart (NEDBEH) (Fregeau et al., 2016; Jordan et al., 2018). The phenotypes seen in individuals with NEDBEH have a strong overlap with those associated with 1p36 deletion syndrome. This suggests that haploinsufficiency of *RERE* contributes to most 1p36 deletion phenotypes, including CHD. Approximately 37% (7/19) of individuals with NEDBEH have VSDs or ASDs. Other forms of CHD documented in individuals with NEDBEH included patent foramen ovale, patent ductus arteriosus, anomalous pulmonary venous return and truncus arteriosus (Fregeau et al., 2016; Jordan et al., 2018).

Mouse embryos that are homozygous for an *Rere* null allele [*om*; c.396+2T>A (NM_001085492.1)] have a more severe phenotype than that associated with NEDBEH in humans. *Rere*^om/om^ mice die at around embryonic day (E) 9.5, and have a spectrum of defects that includes failure of normal cardiac looping, open neural tube defects, irregular partitioning of somites, and fusion of the optic and telencephalic vesicles (Zoltewicz et al., 2004). In contrast, mice that carry an *om* null allele and an *Rere* hypomorphic allele [*eyes3*; c.578T>C (NM_001085492.1), p.Val193Ala] have a spectrum of phenotypes that is similar to that seen in humans with mutations affecting *RERE* (Fregeau et al., 2016; Jordan et al., 2018; Kim and Scott, 2014; Kim et al., 2013). These phenotypes include structural brain anomalies, eye defects, hearing loss and CHDs. Hence, *Rere*^om/eyes3^ mice represent a useful model with which to study the underlying molecular mechanisms by which RERE deficiency affects the development of multiple organs, including the heart.

CHD is present in 100% of *Rere*^om/eyes3^ mice on a C57BL/6 background (Kim et al., 2013). Specific forms of CHD identified in these mice include VSDs, double outlet right ventricle, transposition of the great arteries and aortic arch anomalies. In contrast, CHD is not found in *Rere*^om/eyes3^ embryos on a mixed B6Brd/129S6 background (Kim et al., 2013). This suggests that *Rere*^om/eyes3^ mice could also serve as an effective model with which to identify genetic modifiers of CHD penetrance.

Although RERE clearly plays a crucial role in septal development, the morphogenetic and molecular mechanisms by which RERE deficiency causes septal defects have yet to be determined. During septal development, endocardial cells in a region of the atrioventricular (AV) canal separate from the myocardium. A subset of these cells then undergo epithelial-to-mesenchymal transition (EMT) to form mesenchymal cells, which migrate into the cardiac jelly between the endocardium and the myocardium (Markwald et al., 1981). In the cardiac jelly, these newly formed mesenchymal cells proliferate to fill the AV endocardial cushions that will ultimately give rise to the AV septum and its associated valves (Anderson et al., 2003).

Here, we demonstrate that deficiency of RERE leads to the development of VSDs by decreasing the rate at which endothelial cells in the AV canal undergo EMT, and by decreasing the proliferation of endocardial-derived mesenchymal cells in the AV endocardial cushions. We go on to show that RERE positively regulates the expression of GATA4, a transcription factor implicated in the development of VSDs in humans and mice (Garg et al., 2003; Kostetskii et al., 1999; Yang et al., 2012; Zhang et al., 2008) that positively regulates EMT and promotes mesenchymal proliferation in the AV cushion (Rivera-Feliciano et al., 2006). Tissue-specific ablation of *Rere* in the endocardium leads to hypocellularity of the AV endocardial cushions, defective EMT and VSDs, but does not result in decreased GATA4 expression. This suggests that the cell-autonomous role of RERE in promoting EMT in the endocardium must be mediated by its effects on the expression of proteins other than GATA4.

## RESULTS

### The expression pattern of RERE in the developing heart

Using *in situ* hybridization, Zoltewicz et al. demonstrated that *Rere* is expressed widely in the developing mouse embryo and is specifically expressed in the developing heart at E7.5 and E11.5 (Zoltewicz et al., 2004). We have previously shown that RERE is expressed in the nuclei of cells in the endocardium, myocardium and epicardium of all four chambers of the adult mouse heart (Kim et al., 2013). However, the spatiotemporal expression pattern of RERE in the latter stages of embryonic heart development has not been described in detail.

**Fig. 1.**
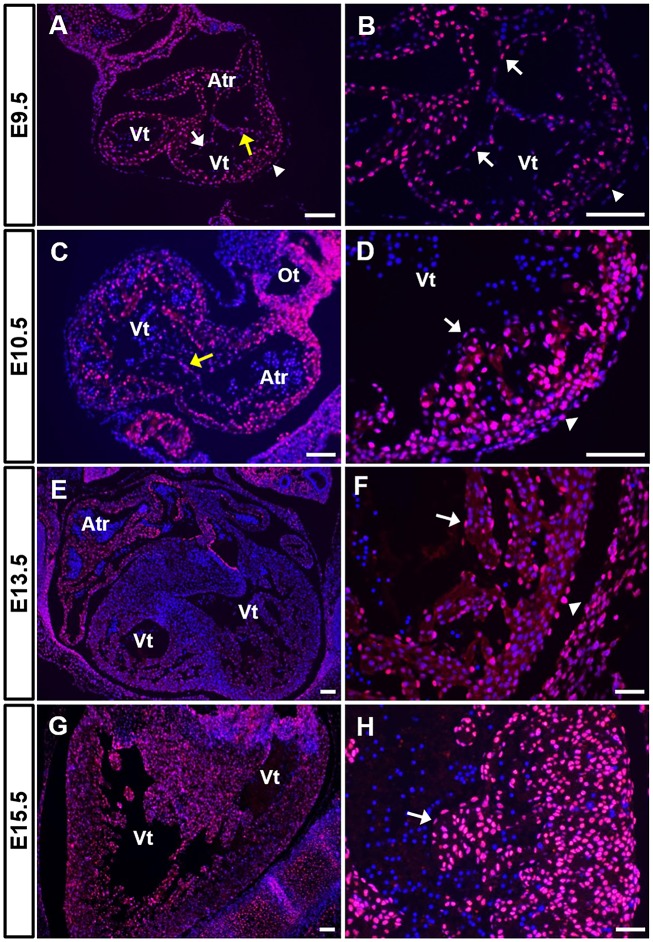
**RERE is expressed in multiple cardiac tissues during development.** (A-H) Cardiac sections were prepared from wild-type C57BL/6 embryos at E9.5 (A,B), E10.5 (C,D), E13.5 (E,F) and E15.5 (G,H) and stained using anti-RERE antibodies. Representative images are shown from the analysis of at least nine sections obtained from each of three or more embryos. RERE-positive cells were labeled in magenta and 4′,6-diamidino-2-phenylindole (DAPI; blue) was used for nuclear staining. Yellow arrows indicate the AV endocardial cushions, white arrows indicate the endocardium and white arrowheads mark the epicardium. Atr, atrium; Ot, outflow tract; Vt, ventricle. Scale bars: 50 µm (B,D), 100 µm (A,C,F,H) or 200 µm (E,G).

To determine the expression pattern of RERE between E9.5 and E15.5, we performed immunohistochemistry on sections from wild-type embryos. Fig. 1 contains representative images obtained from at least nine sections from three or more independent wild-type embryos at each time point. Channel-specific, black and white images for all immunohistochemical panels shown in this paper are available in the Supplementary Information. At E9.5 and E10.5, the looped mouse heart includes a single ventricle, AV canal, atrium and outflow tract. A number of RERE-positive cells were observed in all of these segments at these time points (Fig. 1A-D). RERE-positive cells were detected in the endocardium, the trabecular myocardium, the myocardium and the epicardium (Fig. 1A-D). RERE was also expressed in the mesenchymal cells located in the AV endocardial cushions at E10.5 (Figs 1C and 5A).

At E13.5, the heart consists of four distinct chambers. At this stage, RERE was expressed in the endocardium and the myocardium of all chambers (Fig. 1E,F). RERE-positive cells were also detected in the epicardium (Fig. 1F), in the tricuspid and mitral valves, and in the interventricular septum (Fig. S1A,B). At E15.5, RERE expression was maintained in the endocardium, the myocardium and the epicardium (Fig. 1G,H). RERE-positive cells were broadly detected in the trabecular myocardium and the compact myocardium underlying the epicardium at E15.5 (Fig. 1H). This is in contrast to the relatively few RERE-positive cells that were identified in the myocardium at E13.5 (Fig. 1F).

We also examined the relative level of *Rere* expression in wild-type embryonic hearts at E10.5, E13.5 and E15.5 by quantitative reverse transcription polymerase chain reaction (RT-qPCR). We found that the levels of *Rere* transcripts were decreased in the hearts of E13.5 embryos compared with those in the hearts of E10.5 embryos or E15.5 embryos, consistent with the level of RERE expression seen at each of these stages (Fig. S1C).

### The AV endocardial cushions of RERE-deficient mouse embryos are hypocellular owing to defects in EMT and mesenchymal cell proliferation

**Fig. 2.**
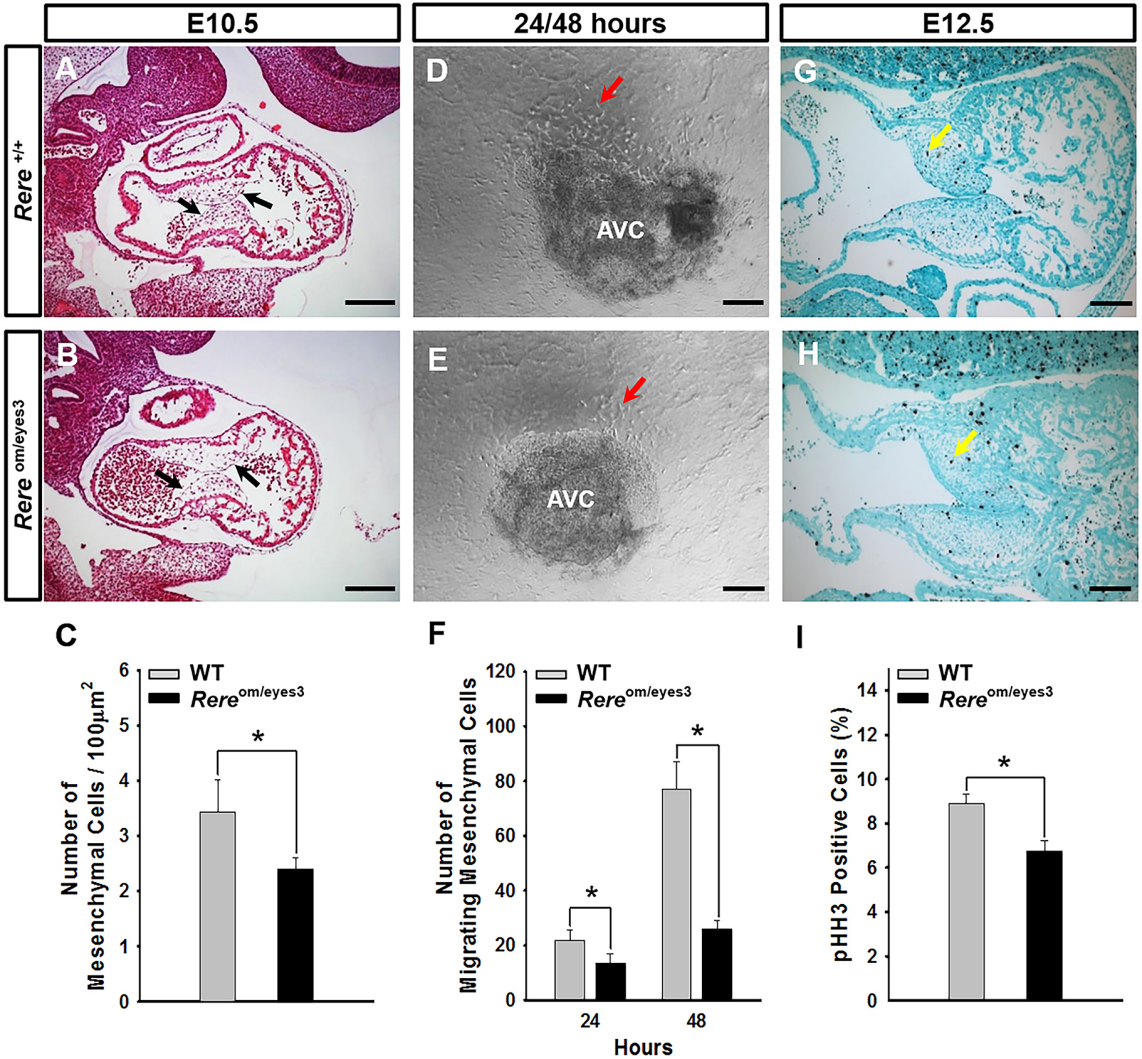
**RERE deficiency leads to the development of hypocellular AV endocardial cushions owing to a defect in EMT and decreased mesenchymal cell proliferation.** (A-C) At E10.5, the mesenchymal cell density in the AV endocardial cushions of *Rere*^om/eyes3^ embryos is significantly reduced in comparison with that of wild-type embryos. Black arrows point to the AV endocardial cushions. Quantification was performed using eight slides containing at least three sections from three independent littermates. Scale bars: 200 µm. (D-F) The AV canal explants isolated from embryos of each genotype were cultured on collagen gels for EMT assays. Migrating mesenchymal cells were counted at 24 h and 48 h. The number of migrating mesenchymal cells generated from AV canal explants prepared from *Rere*^om/eyes3^ embryos was significantly less than the number of those prepared from wild-type embryos. Red arrows indicate migrating mesenchymal cells. AV canal explants prepared from four independent littermates were used for collagen gel culture and quantification. AVC, atrioventricular canal explants. Scale bars: 100 µm. (G-I) At E12.5, the percentage of mitotic cells in the AV endocardial cushions of *Rere*^om/eyes3^ embryos was significantly reduced in comparison with that of wild-type AV endocardial cushions. Yellow arrows indicate mitotic cells in the AV endocardial cushions. Thirty sections obtained from each of three embryos were used for quantification. Scale bars: 100 µm. Data are mean±s.d. Unpaired two-tailed Student's *t*-test was used to determine *P*-values (*P<0.05).

We have previously shown that VSDs are present in ∼67% of *Rere*^om/eyes3^ embryos on a C57BL/6 background at E15.5 (Kim et al., 2013). Because the mesenchymal cells of the AV endocardial cushions will ultimately form the AV septum and its associated valves (Anderson et al., 2003), we looked for defects in the development of the AV endocardial cushions in *Rere*^om/eyes3^ embryos. When we examined sagittal sections through the AV endocardial cushions of *Rere*^om/eyes3^ embryos at E10.5, we found that the endocardium had successfully separated from the underlying myocardium and that the sizes of the AV endocardial cushions were comparable to those of wild-type control littermates (Fig. 2A,B). However, the AV endocardial cushions of the *Rere*^om/eyes3^ embryos were hypocellular, with the number of mesenchymal cells/area in the AV endocardial cushions of *Rere*^om/eyes3^ embryos being significantly lower (*P*<0.05) than that observed in the AV endocardial cushions of wild-type control littermates (Fig. 2C).

To determine whether a defect in EMT could be contributing to the hypocellularity of the AV endocardial cushions of *Rere*^om/eyes3^ embryos, we performed collagen gel AV canal explant studies. Briefly, the AV canals of *Rere*^om/eyes3^ embryos and wild-type littermate controls were harvested at E9.5 and cultured on a collagen gel (Fig. 2D,E). We found that the number of migrating mesenchymal cells generated from *Rere*^om/eyes3^ AV canal explants at 24 h and 48 h was significantly reduced (*P*<0.05) in comparison with the number generated from the AV canal explants of wild-type control littermates (Fig. 2F). This suggests that a defect in EMT is a likely contributor to the hypocellularity seen in *Rere*^om/eyes3^ AV endocardial cushions.

After undergoing EMT, endothelial-derived mesenchymal cells proliferate to fill the AV endocardial cushions. To determine whether a reduction in mesenchymal cell proliferation, or an increase in mesenchymal cell apoptosis, could also be contributing to the development of VSDs in RERE-deficient embryos, we stained sagittal sections from E12.5 *Rere*^om/eyes3^ embryos and wild-type control littermates with anti-phospho-histone H3 (a marker of proliferation) and anti-cleaved caspase-3 (a marker of apoptosis), respectively. The percentage of *Rere*^om/eyes3^ AV cushion cells that were positive for phospho-histone H3 was significantly decreased (*P*<0.05) when compared with that of wild-type littermate controls (Fig. 2G-I). Cleaved caspase-3-positive cells were only rarely identified in the AV canals of *Rere*^om/eyes3^ embryos and wild-type littermate controls (data not shown). This suggests that RERE deficiency leads to a defect in mesenchymal cell proliferation, but has no significant effect on mesenchymal cell apoptosis within the developing AV cushion.

### Generation and testing of an *Rere* conditional knockout allele

To determine the effects of RERE deficiency in specific cardiac tissues, we used a recombineering strategy (Liu et al., 2003) to generate an *Rere* conditional allele (*Rere* flox) in which the second coding exon of *Rere* – the same exon that is skipped in the *om* null allele – is flanked by loxP sites (Fig. S2). *Rere*^flox/flox^ mice were generated in expected numbers in heterozygous crosses, were viable and fertile, and had no discernable abnormal phenotypes.

**Fig. 3.**
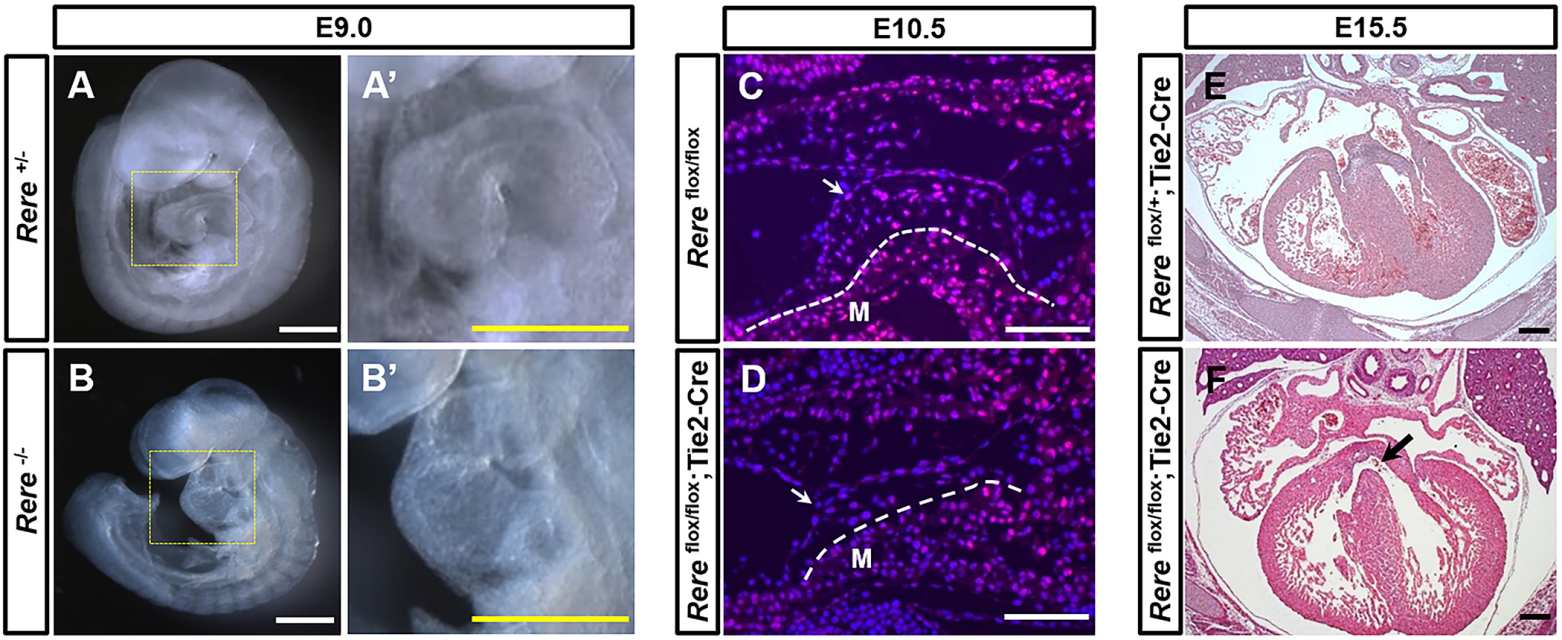
**Ablation of *Rere* in the endocardium leads to the development of VSDs.** To test the ability of the *Rere* flox allele to generate a null allele after exposure to Cre, we generated a line of *Rere*^+/−^ mice by crossing *Rere*^flox/flox^ mice with mice carrying an Hprt-Cre allele (Tang et al., 2002). (A,A′) As expected, E9.0 *Rere*^+/−^ embryos had normal cardiac looping. (B,B′) E9.0 *Rere*^−/−^ embryos showed failure of cardiac looping, as previously documented in embryos that are homozygous for the *om* null allele (Zoltewicz et al., 2004). Scale bars: 0.5 mm. (C,D) Sagittal cardiac sections were prepared from E10.5 *Rere*^flox/flox^ and Tie2-Cre; *Rere*^flox/flox^ embryos and stained with anti-RERE antibodies. *Rere*^flox/flox^;Tie2-Cre embryos showed a tissue-specific decrease in RERE immunoreactivity in the endocardium and in the mesenchymal cells of the AV endocardial cushions when compared with *Rere*^flox/flox^ littermate control embryos. Arrows indicate the endocardium; dashed lines mark the boundary of the myocardium and the AV endocardial cushions. M, myocardium. Scale bars: 100 µm. Representative images are shown from the analysis of at least nine sections obtained from each of three embryos. (E) E15.5 *Rere*^*flox/+*^;Tie2-Cre embryos have normal ventricular septums. (F) Approximately 33% (2/6) of *Rere*^flox/flox^;Tie2-Cre embryos at E15.5 have VSDs (black arrow). Scale bars: 200 µm.

To test the ability of the *Rere* flox allele to generate a null allele after exposure to Cre, we established a line of *Rere*^+/−^ mice by crossing *Rere*^flox/flox^ mice with mice carrying an Hprt-Cre allele (Tang et al., 2002). As expected *Rere*^+/−^ embryos (Fig. 3A) and mice were phenotypically normal. These heterozygous mice were then crossed with each other to generate *Rere*^−/−^ embryos, which died at around E9.5, and had unlooped hearts (Fig. 3B) and neural tube defects (Fig. S3A,B), as previously documented in *Rere*^om/om^ embryos (Zoltewicz et al., 2004). As a further test, we performed immunohistochemistry on sagittal sections from E9.5 *Rere*^+/−^ and *Rere*^−/−^ embryos. RERE-positive cells were broadly detected in *Rere*^+/−^ embryonic hearts at E9.5, whereas RERE-positive cells were not visible in *Rere*^−/−^ embryonic hearts at the same time point (Fig. S3C,D). This suggests that Cre-mediated recombination of the loxP sites of the *Rere* flox allele generates an *Rere* null allele. This also suggests that the anti-RERE antibody used in this study is highly specific for RERE.

As a further test of the *Rere* flox allele, we generated *Rere*^flox/flox^;*Nkx2-5*^Cre/+^ embryos on a mixed B6/129S6 background. The knock-in *Nkx2-5* Cre allele renders these embryos haploinsufficient for *Nkx2-5* and is expected to cause ablation of *Rere* in the cardiac progenitor cells of the cardiac crescent starting as early as E7.5 (Moses et al., 2001). In keeping with this expected outcome, we demonstrated reduced RERE immunoreactivity in the myocardium of *Rere*^flox/flox^;*Nkx2-5*^Cre/+^ embryos in comparison with *Rere*^flox/flox^ littermate control embryos at E10.5 (Fig. S3E,F). At E15.5, 100% (3/3) of the *Rere*^flox/flox^;*Nkx2-5*^Cre/+^ embryos were found to have double outlet right ventricle/overriding aorta and VSDs (Fig. S4, Table S1). In contrast, cardiac malformations were not identified in *Rere*^flox/flox^ (*n*=3) or *Rere*^flox/+^;*Nkx2-5*^Cre/+^ littermate embryos (*n*=3) at E15.5. This provides evidence of tissue-specific recombination of the *Rere* flox allele in the presence of Cre expression and confirms the role of RERE in cardiac development.

### Ablation of *Rere* in the endocardium causes VSDs through its effects on EMT

Based on the abnormal levels of EMT seen in *Rere*^om/eyes3^ embryos, we hypothesized that *Rere* plays a crucial role in the endocardium during AV canal formation. To test this hypothesis, we generated *Rere*^flox/flox^;Tie2-Cre embryos. The transgenic Tie2-Cre allele does not affect expression of the *Tie2* (also known as *Tek*) gene but is expected to cause ablation of *Rere* in endothelial cells, including the endocardium (Kisanuki et al., 2001). As expected, immunohistochemical staining of sections obtained from these *Rere*^flox/flox^;Tie2-Cre embryos at E10.5 showed a tissue-specific decrease in RERE immunoreactivity in the endocardium and in the endothelial-derived mesenchymal cells of the AV endocardial cushions, when compared with *Rere*^flox/flox^ littermate control embryos, whereas RERE immunoreactivity was maintained in the myocardium (Fig. 3C,D).

We then analyzed the cardiac structure of E15.5 *Rere*^flox/flox^;Tie2-Cre embryos and *Rere*^flox/+^;Tie2-Cre controls in serial transverse sections on a CHD-resistant B6/129S6 background and a CHD-permissive C57BL/6 background. On a mixed B6/129S6 background, no cardiac anomalies were identified among *Rere*^flox/flox^;Tie2-Cre embryos at E15.5 (data not shown). In contrast, perimembranous VSDs were identified in 33% (2/6) of *Rere*^flox/flox^;Tie2-Cre embryos on a C57BL/6 background (Fig. 3E,F; Table S2). In contrast to the majority of VSDs observed in *Rere*^om/eyes3^ on a C57BL/6 background, these VSDs were not associated with conotruncal anomalies, such as double outlet right ventricle/overriding aorta. As expected, no cardiac defects were identified in *Rere*^flox/+^;Tie2-Cre littermate control embryos (*n*=6) (Fig. 3E; Table S2).

**Fig. 4.**
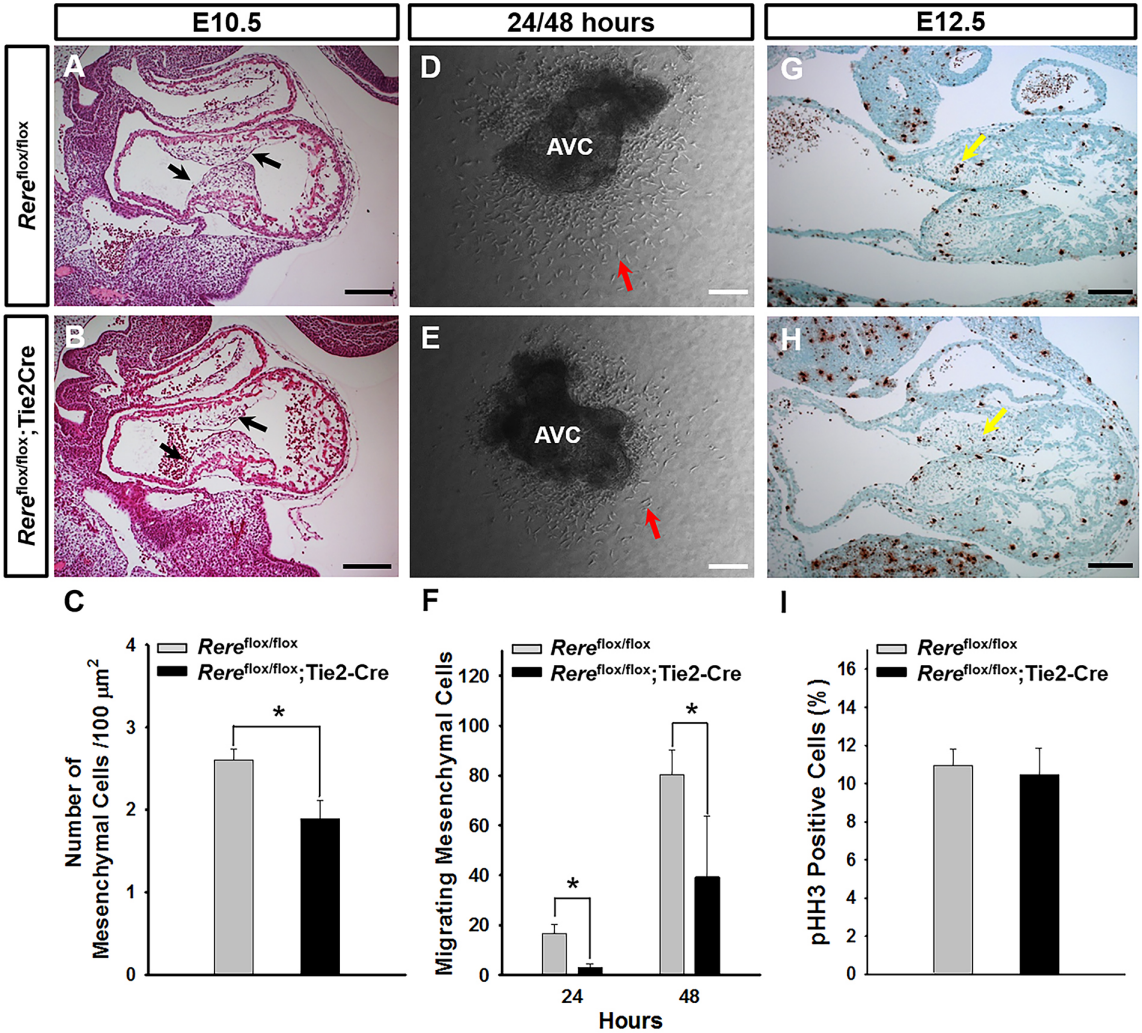
**Ablation of *Rere* in the endocardium leads to the development of hypocellular AV endocardial cushions and defective EMT.** (A-C) At E10.5, the mesenchymal cell density in the AV endocardial cushions of *Rere*^flox/flox^;Tie2-Cre embryos is significantly reduced in comparison with that of *Rere*^flox/flox^ embryos. Black arrows point to the AV endocardial cushions. Quantification was performed using eight slides containing at least three sections from five independent littermates. Scale bars: 200 µm. (D-F) The AV canal explants isolated from the hearts of each of the *Rere*^flox/flox^;Tie2-Cre and *Rere*^flox/flox^ embryos were cultured on collagen gels. Migrating mesenchymal cells were counted at 24 h and 48 h. The number of migrating mesenchymal cells generated from AV canal explants of *Rere*^flox/flox^;Tie2 Cre embryos is significantly reduced in comparison with that of *Rere*^flox/flox^ embryos. Red arrows indicate migrating mesenchymal cells. AV canal explants prepared from five independent littermates were used for collagen gel culture and quantification. AVC, atrioventricular canal explants. Scale bars: 100 µm. (G-I) At E12.5, the percentage of mitotic cells in the AV endocardial cushions of *Rere*^flox/flox^;Tie2 Cre embryos was comparable to that of *Rere*^flox/flox^ embryos. Yellow arrows indicate mitotic cells in the AV endocardial cushions. Twenty-one sections obtained from each of three embryos were used for quantification. Scale bars: 100 µm. Data are mean±s.d. Unpaired two-tailed Student's *t*-test was used to determine *P*-values (*P<0.05).

To determine whether ablation of *Rere* in the endocardium caused hypocellularity of the endocardial cushions, we then examined sagittal sections through the AV canals of *Rere*^flox/flox^;Tie2-Cre embryos and *Rere*^flox/flox^ control embryos at E10.5. We found that the endocardium had successfully separated from the underlying myocardium in *Rere*^flox/flox^;Tie2-Cre embryos and that the sizes of the AV endocardial cushions in these embryos were comparable to those of their *Rere*^flox/flox^ littermate controls (Fig. 4A,B). However, the AV endocardial cushions of the *Rere*^flox/flox^;Tie2-Cre embryos were hypocellular, with the number of mesenchymal cells/area in the AV endocardial cushions of *Rere*^flox/flox^;Tie2-Cre embryos being significantly lower (*P*<0.05) than that observed in the AV endocardial cushions of their *Rere*^flox/flox^ littermates (Fig. 4C).

Because hypocellularity of the AV endocardial cushions was associated with abnormal EMT in *Rere*^om/eyes3^ embryos, we evaluated EMT levels in *Rere*^flox/flox^;Tie2-Cre embryos using AV canal explant cultures (Fig. 4D,E). At 24 h and 48 h, the AV canal explants of *Rere*^flox/flox^;Tie2-Cre embryos generated significantly fewer (*P*<0.05) migrating mesenchymal cells than explants from their *Rere*^flox/flox^ control littermates (Fig. 4F). This suggests that ablation of *Rere* in the endocardium causes decreased levels of EMT, contributing to the hypocellularity of the endocardial cushions and VSDs seen in *Rere*^flox/flox^;Tie2-Cre embryos.

To determine whether a reduction in mesenchymal cell proliferation could also be contributing to the development of VSDs in *Rere*^flox/flox^;Tie2-Cre embryos, we stained sagittal sections from E12.5 *Rere*^flox/flox^;Tie2-Cre embryos and their *Rere*^flox/flox^ control littermates with anti-phospho-histone H3. In contrast to the decreased mesenchymal cell proliferation rate seen in *Rere*^om/eyes3^ embryos, the percentage of *Rere*^flox/flox^;Tie2-Cre AV cushion cells that were positive for phospho-histone H3 was comparable to that of *Rere*^flox/flox^ littermate controls (Fig. 4G-I). This suggests that a decrease in mesenchymal cell proliferation rate is unlikely to be contributing to the hypocellularity of the endocardial cushions and VSDs in *Rere*^flox/flox^;Tie2-Cre embryos.

### RERE promotes the expression of GATA4 in the endocardium of the AV canal

RERE has been shown to positively regulate retinoic acid signaling, and loss of RERE function leads to a dramatic decrease in retinoic acid signaling in the developing heart (Vilhais-Neto et al., 2010; Zoltewicz et al., 2004). GATA4 – a retinoic acid-inducible transcription factor implicated in the development of VSDs in humans and mice – has been shown to function in the endocardium to promote EMT, and to promote mesenchymal cell proliferation (Jay et al., 2007; Kostetskii et al., 1999; Pu et al., 2004; Rivera-Feliciano et al., 2006; Yang et al., 2012; Zhang et al., 2008). Similarities between the cardiac phenotypes seen in RERE-deficient and GATA4-deficient embryos led us to hypothesize that RERE functions, at least in part, to promote the expression of GATA4 in the endocardium of the AV canal.

**Fig. 5.**
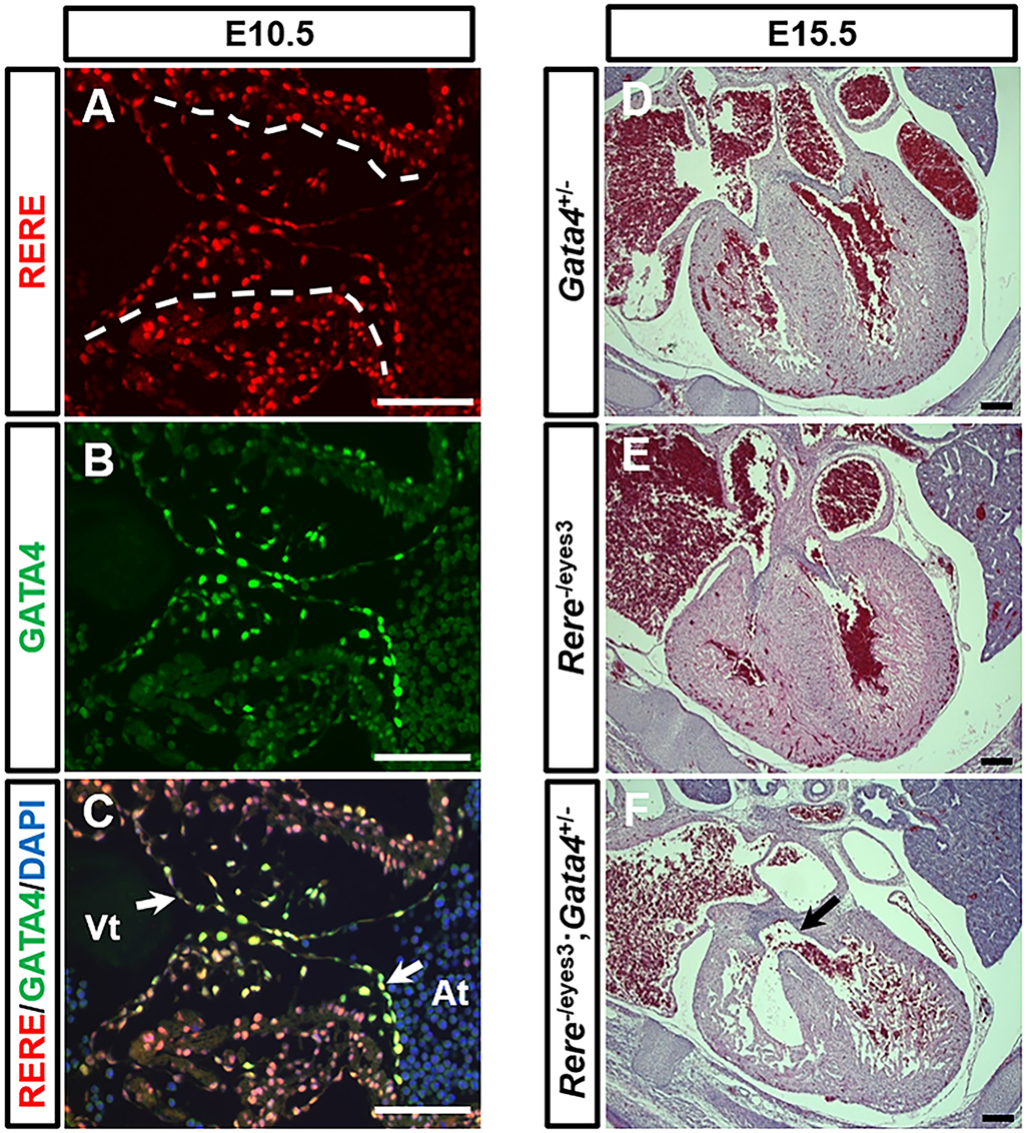
**RERE and GATA are co-expressed in the AV canal, and *Rere* and *Gata4* interact genetically in the development of CHD.** (A-C) Sections of wild-type embryos were prepared at E10.5 and stained with anti-RERE and anti-GATA4 antibodies. RERE (red) and GATA4 (green) were colocalized in the endocardium and mesenchymal cells of the AV endocardial cushions. Dashed lines indicate the boundary between the AV endocardial cushions and the myocardium; white arrows indicate the endocardium of the AV cushion. Representative images are presented from sections obtained from four wild-type embryos. Atr, atrium; Vt, ventricle. Scale bars: 100 µm. (D,E) *Gata4*^+/−^ and *Rere*^−/eyes3^ embryos on a mixed B6/129S6 background have normal ventricular septums at E15.5. (F) E15.5 *Rere*^−/eyes3^;*Gata4*^+/−^ embryos on a mixed B6/129S6 background have several types of CHD including perimembranous VSDs (arrow). Scale bars: 200 µm.

To test this hypothesis, we first sought to determine whether RERE and GATA4 are co-expressed in the developing AV canal using immunohistochemistry. In wild-type embryos, RERE-positive cells were located in the endocardium, the mesenchymal cells of the AV cushion and the myocardium at E10.5 (Fig. 5A). However, the majority of GATA4-expressing cells were seen in the endocardium and the mesenchymal cells of the AV endocardial cushions at E10.5 (Fig. 5B). A number of cells in the endocardium and mesenchymal cells of the AV endocardial cushions were clearly positive for both RERE and GATA4 (Fig. 5C).

We then sought to determine whether *Rere* and *Gata4* interact genetically in the development of CHD. As previously described, CHD is not found in *Rere*^om/eyes3^ embryos on a mixed B6/129S6 background, whereas *Rere*^om/eyes3^ embryos on a C57BL/6 background develop CHD (Kim et al., 2013). *Gata4* follows a similar pattern, with CHDs being absent in *Gata4* haploinsufficient mice on mixed backgrounds, but present in a subset of *Gata4*^+/−^ mice on a C57BL/6 background (Jay et al., 2007; Kuo et al., 1997; Molkentin et al., 1997; Pu et al., 2004; Rajagopal et al., 2007). With this in mind, we determined the cardiac phenotype of *Rere*^−/eyes3^;*Gata4*^+/−^ embryos at E15.5 on the CHD-resistant B6/129S6 background on which *Rere*^−/eyes3^ and *Gata4*^+/−^ embryos would not be expected to have CHD (Fig. 5D,E). On this background, *Rere*^−/eyes3^, *Gata4*^+/−^ and *Rere*^−/eyes3^;*Gata4*^+/−^ embryos could not be distinguished based on their gross morphology at E15.5. However, 100% (3/3) of *Rere*^−/eyes3^;*Gata4*^+/−^ embryos developed CHD (Fig. 5F; Table S3). Specific defects observed in one or more *Rere*^−/eyes3^;*Gata4*^+/−^ embryos included VSD, preductal coarctation of the aorta, right-sided aortic arch, retroesophageal ductus, aberrant left subclavian artery and abnormal tricuspid valve (Fig. 5F; Fig. S5). In contrast, *Rere*^−/eyes3^ and *Gata4*^+/−^ littermate controls (*n*=3 of each genotype) did not develop CHD, with the exception of one *Rere*^−/eyes3^ and one *Gata4*^+/−^ embryo that had aberrant right subclavian arteries (Table S3). This suggests that *Rere* and *Gata4* interact genetically in the development of CHD.

**Fig. 6.**
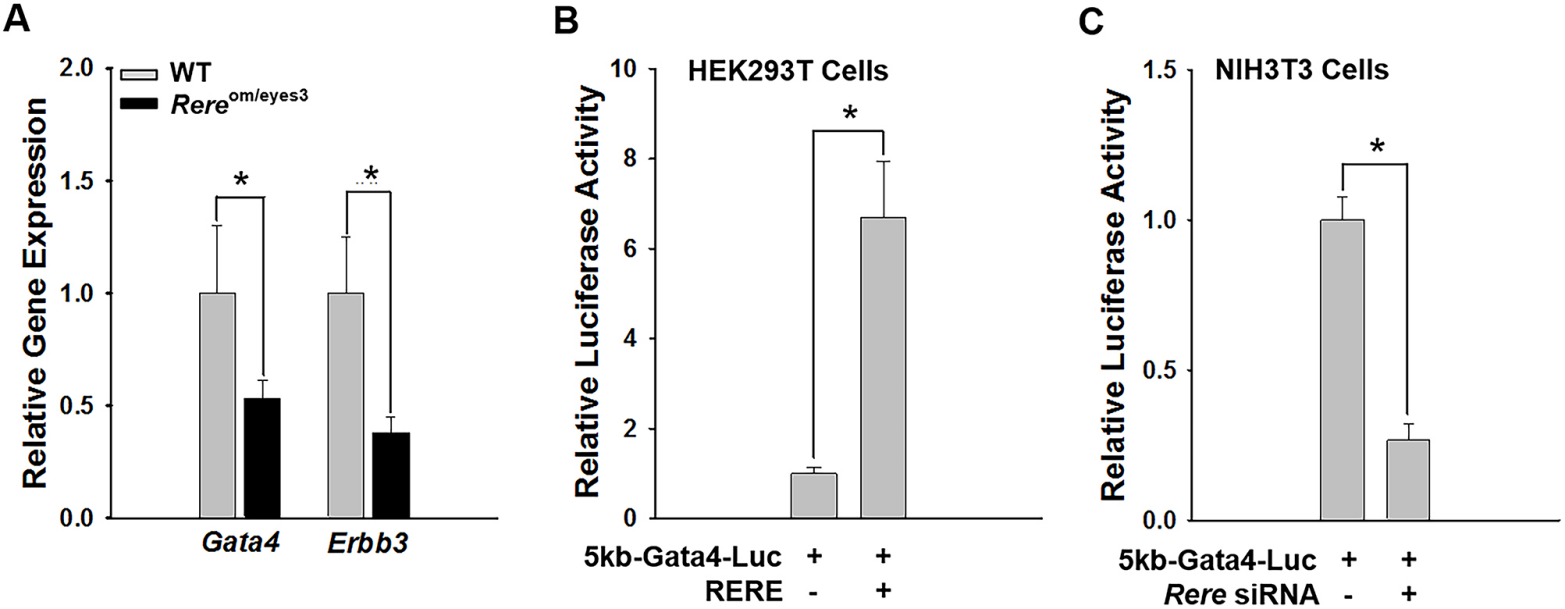
**RERE regulates the transcription of *Gata4*.** (A) RT-qPCR analyses were performed using mRNA extracted from the hearts of wild-type and *Rere*^om/eyes3^ embryos at E10.5. The relative amounts of *Gata4* and *Erbb3* transcripts were significantly reduced in the hearts of *Rere*^om/eyes3^ embryos compared with those of wild-type embryos. Three independent RT-qPCR analyses were performed using mRNA prepared from the hearts of three different littermates. (B) A firefly luciferase reporter gene fused with a previously described 5 kb promoter of *Gata4* (5kb-Gata4-Luc) (Mazaud Guittot et al., 2007) was transfected into HEK293 T cells with/without an *RERE*-expressing vector. Luciferase activity was normalized by cotransfection with a Renillar luciferase plasmid. Luciferase activity driven by the 5 kb *Gata4* promoter was increased by overexpression of RERE. Experiments were performed in triplicate. (C) A 5kb-Gata4-Luc plasmid was transfected into NIH3T3 cells with a nontargeting siRNA pool or an *Rere* siRNA pool. Luciferase activity was normalized by cotransfection with a Renillar luciferase plasmid. NIH3T3 cells transfected with the *Rere* siRNA pool showed decreased luciferase activity when compared with those transfected with a nontargeting siRNA pool. Luciferase experiments were performed in triplicate. In all graphs, data are mean±s.d. Unpaired two-tailed Student's *t*-test was used to determine *P*-values (*P<0.05).

Because RERE is a nuclear receptor co-regulator, it is reasonable to assume that it functions within cells to modulate transcription (Wang et al., 2008; Zoltewicz et al., 2004). This assumption is consistent with previously published data in which RERE has been shown to activate and inhibit the transcription of specific target genes (Kumar and Duester, 2014; Vilhais-Neto et al., 2010). To determine whether RERE regulates the expression of *Gata4* in the heart, we compared the level of *Gata4* transcripts in E10.5 hearts of *Rere*^om/eyes3^ and wild-type littermate control embryos by RT-qPCR. We found that the levels of *Gata4* transcripts were significantly (*P*<0.05) decreased in hearts harvested from *Rere*^om/eyes3^ embryos compared with those from wild-type littermate controls (Fig. 6A). GATA4 promotes EMT in the AV canal by positively regulating *Erbb3* expression (Rivera-Feliciano et al., 2006). Consistent with this mechanism, we also found decreased *Erbb3* transcript levels in E10.5 hearts from *Rere*^om/eyes3^ embryos compared with those from their wild-type control littermates (*P*<0.05; Fig. 6A). In contrast, no change was seen in the transcript levels of other EMT-related genes, including *Bmp2*, *Bmp4*, *Bmp5*, *Nfatc1*, *Snai1*, *Snai2* and *Notch1* (Fig. S6).

To determine whether RERE can regulate the transcriptional activity of the *Gata4* promoter, we performed luciferase assays in HEK293T cells using a vector containing a luciferase reporter gene fused with a previously described 5 kb *Gata4* promoter (5kb-Gata4-Luc) in the presence or absence of an *RERE* expression vector (Mazaud Guittot et al., 2007). As expected, mock vector transfected HEK293T cells had undetectable levels of RERE protein. In contrast, RERE protein was clearly detected in HEK293T cells transfected with the *RERE* expression vector (Fig. S7A). The luciferase activity of the 5 kb *Gata4* promoter increased significantly (*P*<0.05) in HEK293T cells that overexpressed RERE (Fig. 6B).

To determine whether loss of RERE can also affect the transcriptional activity of the *Gata4* promoter, we performed luciferase assays in NIH3T3 cells that endogenously express RERE. Briefly, NIH3T3 cells transfected with the 5kb-Gata4-Luc plasmid were transfected with an *Rere* short interfering RNA (siRNA) pool or a nontargeting siRNA pool. As expected, NIH3T3 cells transfected with the *Rere* siRNA pool showed decreased levels of RERE protein expression compared with NIH3T3 cells transfected with the nontargeting siRNA pool (Fig. S7B). NIH3T3 cells transfected with the *Rere* siRNA pool had significantly decreased (*P*<0.05) luciferase activity when compared with NIH3T3 cells transfected with the nontargeting siRNA pool (Fig. 6C).

Because RERE has been shown to positively regulate retinoic acid signaling during development, and *Gata4* is a retinoic acid-inducible transcription factor, we hypothesized that treatment with retinoic acid might stimulate transcription from the 5kb-Gata4-Luc plasmid in NIH3T3 cells (Arceci et al., 1993; Kostetskii et al., 1999; Vilhais-Neto et al., 2010; Zoltewicz et al., 2004). However, the addition of 0.25, 0.5, 1.0, 2.0 and 3.0 µM all-trans retinoic acid did not result in increased luciferase activity compared with vehicle (Fig. S8).

### GATA4 protein levels are decreased in the AV canals of *Rere*^om/eyes3^ embryos but not in *Rere*^flox/flox^;Tie2-Cre embryos

**Fig. 7.**
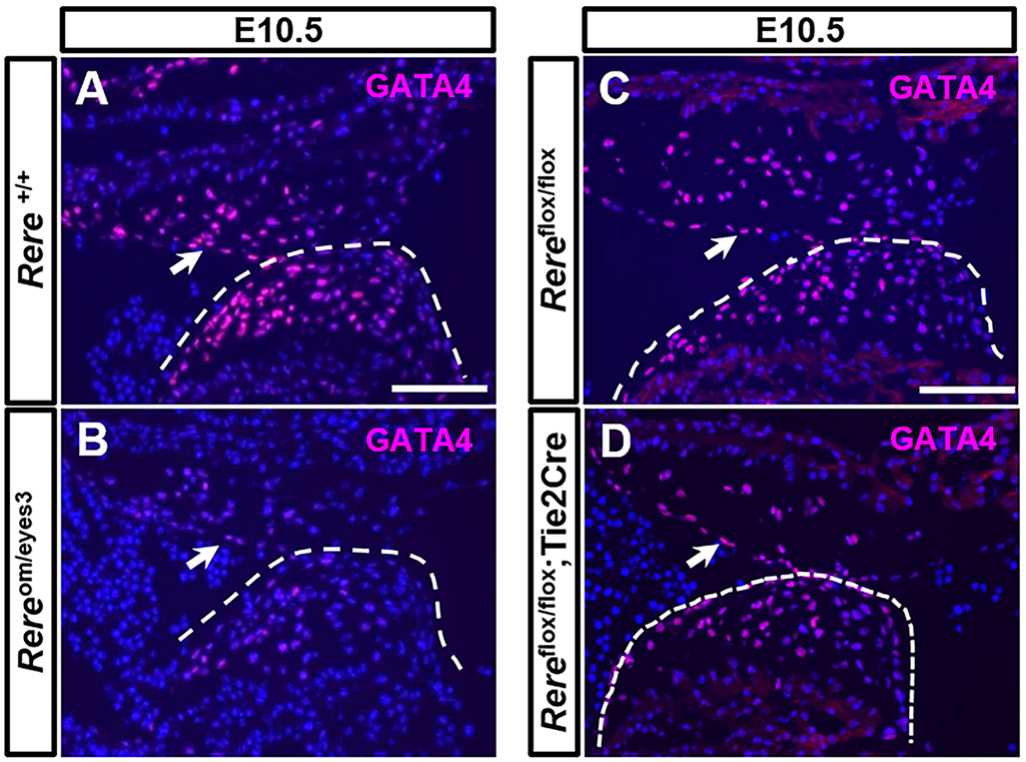
**RERE regulates the expression of GATA4 in the AV canal.** (A,B) GATA4 was visualized with anti-GATA4 antibodies on sections through the AV canals of wild-type embryos and *Rere*^om/eyes3^ embryos. The immunoreactivity of GATA4 in the wild-type AV endocardial cushions was stronger than that observed in *Rere*^om/eyes3^ AV endocardial cushions. (C,D) In contrast, the levels of GATA4 expression were comparable in the AV canals of *Rere*^flox/flox^ and *Rere*^flox/flox^;Tie2-Cre embryos. These results suggest that decreased expression of RERE in cells other than the endocardium play a role in regulating the expression of GATA4 in the AV canal. Arrows and dashed lines indicate the AV endocardial cushions. Representative images are shown from the analysis of sections from three embryos of each genotype. Scale bars: 100 µm.

To confirm that deficiency of RERE leads to decreased expression of GATA4 protein, we performed GATA4 immunohistochemical analysis on sagittal sections through the AV canals of E10.5 *Rere*^om/eyes3^ embryos and wild-type littermate controls. Cells with strong immunoreactivity for GATA4 were easily detected in the endocardial and mesenchymal cells of the AV endocardial cushions of wild-type embryos at this time point (Fig. 7A). In comparison, the level of GATA4 immunoreactivity in the endocardial and mesenchymal cells of the AV endocardial cushions of *Rere*^om/eyes3^ embryos was reduced (Fig. 7B). This suggests that RERE deficiency leads to decreased expression of GATA4 in the AV canal.

We repeated these studies, comparing the expression of GATA4 in the AV canals of E10.5 *Rere*^flox/flox^;Tie2-Cre embryos and *Rere*^flox/flox^ littermate controls. Surprisingly, the level of GATA4 expression in the AV canal was comparable between embryos of these genotypes (Fig. 7C,D).

## DISCUSSION

In humans, mutations affecting *RERE* cause a syndromic form of CHD, and *RERE* haploinsufficiency is a major contributor to the phenotypes associated with proximal deletions of chromosome 1p36 (Fregeau et al., 2016; Jordan et al., 2018). Septal defects are the most common type of CHD associated with RERE deficiency in humans. These septal defects are usually not found in conjunction with conotruncal anomalies. Here, we have used a variety of RERE-deficient mice – including mice carrying a newly generated *Rere* conditional allele – to discover the morphogenetic and molecular pathways by which RERE deficiency leads to the development of VSDs.

### Defective EMT and a decrease in mesenchymal cell proliferation lead to the development of hypocellular AV endocardial cushions in RERE-deficient mice

During AV canal development, signals from the myocardium induce expansion of the cardiac jelly between the myocardium and the endocardium, which results in inward swelling of the AV canal lumen. After separation of the endocardium, a subset of endocardial cells undergo EMT and are transformed into mesenchymal cells, which migrate into the cardiac jelly and proliferate to fill the AV endocardial cushions (Combs and Yutzey, 2009; Eisenberg and Markwald, 1995; Lin et al., 2012). In the AV canals of *Rere*^om/eyes3^ embryos, the endocardium separates properly from the underlying myocardium. However, the AV endocardial cushions of these embryos have significantly decreased (*P*<0.05) mesenchymal cell density compared with those of wild-type embryos. Because mesenchymal cells in the AV endocardial cushions ultimately form part of the ventricular septum (Anderson et al., 2003), this decrease in mesenchymal cell density is likely to be the underlying cause of the nonconotruncal VSDs observed in RERE-deficient mice and individuals with mutations affecting *RERE* (Fregeau et al., 2016; Jordan et al., 2018; Kim et al., 2013).

Because the AV canals of *Rere*^om/eyes3^ embryos are hypocellular, and RERE is expressed in the endocardial cells of the AV canal, we hypothesized that RERE deficiency could cause a defect in EMT. Our studies revealed a significant reduction (*P*<0.05) in the number of migrating mesenchymal cells generated from AV canal explants obtained from *Rere*^om/eyes3^ embryos compared with those obtained from wild-type controls. These results suggest that RERE deficiency leads to a defect in EMT which, in turn, contributes to the development of hypocellular AV endocardial cushions.

Because RERE continues to be expressed in the mesenchymal cells of the AV endocardial cushions, we hypothesized that a decrease in mesenchymal cell proliferation or an increase in mesenchymal cell apoptosis could also contribute to the hypocellularity of the AV endocardial cushions observed in *Rere*^om/eyes3^ embryos. Immunohistochemical evaluations revealed significantly decreased (*P*<0.05) numbers of phospho-histone H3-positive cells in the E12.5 AV endocardial cushions of *Rere*^om/eyes3^ embryos compared with those of wild-type control embryos. This led us to conclude that RERE deficiency also causes a decrease in mesenchymal cell proliferation, which contributes to the hypocellularity of the AV endocardial cushions. In contrast, the numbers of anti-cleaved caspase-3-positive cells in the AV canals of *Rere*^om/eyes3^ and wild-type embryos was not statistically different, leading us to conclude that RERE deficiency does not have a significant impact on mesenchymal cell apoptosis in the AV canal.

### RERE deficiency in the endocardium causes abnormal EMT and VSDs

The effects of RERE on EMT and mesenchymal cell proliferation led us to hypothesize that ablation of *Rere* in endocardial cells would lead to the development of VSDs. We tested this hypothesis by ablating *Rere* in endothelial cells, including the endocardium, using a transgenic Tie2-Cre allele. On a C57BL/6 background, ∼33% (2/6) of *Rere*^flox/flox^;Tie2-Cre embryos developed nonconotruncal, perimembranous VSDs.

The size and morphology of the AV endocardial cushions was comparable between *Rere*^flox/flox^;Tie2-Cre embryos and *Rere*^flox/flox^ embryos. However, at E10.5, the AV endocardial cushions of *Rere*^flox/flox^;Tie2-Cre embryos showed hypocellularity in comparison with those of *Rere*^flox/flox^ embryos. In addition, the number of migrating mesenchymal cells was significantly decreased (*P*<0.05) in the AV canal explants of *Rere*^flox/flox^;Tie2-Cre embryos when compared with that in the AV canal explants of *Rere*^flox/flox^ embryos. These results led us to conclude that ablation of *Rere* in the endocardium leads to defective EMT and contributes to development of nonconotruncal VSDs.

The proliferation rate of mesothelial cells in the AV canals of *Rere*^flox/flox^;Tie2-Cre embryos was similar to that of *Rere*^flox/flox^ littermate controls. This suggests that the decreased levels of mesenchymal cell proliferation observed in *Rere*^om/eyes3^ embryos requires loss of RERE expression in cells other than the endocardium. Maintenance of mesenchymal cell proliferation in the AV canal might explain why only a subset of *Rere*^flox/flox^;Tie2-Cre embryos develop VSDs, despite the effect of endocardial RERE depletion on EMT levels in the AV canal.

### RERE modulates the transcription of *Gata4*

RERE is a nuclear receptor co-regulator (Vilhais-Neto et al., 2010; Wang et al., 2008; Zoltewicz et al., 2004). This suggests that the defects seen in RERE-deficient mice are likely to be caused by dysregulation of RERE target genes. Several lines of evidence led us to hypothesize that *Gata4* is a downstream target of RERE in the AV canal. First, RERE is known to control retinoic acid signaling in the heart and GATA4 is a cardiac-expressed retinoic acid-inducible transcription factor (Arceci et al., 1993; Kostetskii et al., 1999). Second, the phenotypes manifested by RERE- and GATA4-deficient embryos are similar, with both having hypocellular AV canals and VSDs as a result of abnormal EMT and decreased mesenchymal cell proliferation (Jay et al., 2007; Kostetskii et al., 1999; Pu et al., 2004; Rivera-Feliciano et al., 2006; Yang et al., 2012; Zhang et al., 2008). Third, the ablation of either *Rere* or *Gata4* in the endocardium using a Tie2-Cre allele leads to the development of VSDs (Rivera-Feliciano et al., 2006).

To test this hypothesis, we first confirmed that RERE and GATA4 were co-expressed in the endocardium and mesenchymal cells of the AV canal. We then demonstrated that *Rere* and *Gata4* interact genetically in the development of CHD, with E15.5 *Rere*^−/eyes3^;*Gata4*^+/−^ embryos on a B6/129S6 background having VSDs, double outlet right ventricle, and tricuspid valve and aortic arch anomalies not seen in *Rere*^−/eyes3^ and *Gata4*^+/−^ littermate controls. We went on to show a decrease in the abundance of *Gata4* transcripts and GATA4 protein in the hearts of *Rere*^om/eyes3^ embryos compared with wild-type controls. RT-qPCR studies also revealed decreased levels of *Erbb3* transcripts in the hearts of *Rere*^om/eyes3^ embryos compared with those seen in wild-type littermate controls. Although we cannot exclude the possibility that RERE regulates *Erbb3* transcription, we note that GATA4 has been previously shown to promote EMT in the AV canal by positively regulating *Erbb3* expression (Rivera-Feliciano et al., 2006). Hence, it is likely that this is a secondary effect of decreased GATA4 expression.

We then performed luciferase assays in which we showed that overexpression of RERE can increase the transcriptional activity from a 5 kb *Gata4* promoter in HEK293T cells. We also demonstrated that knockdown of *Rere* in NIH3T3 cells that endogenously express RERE led to decreased transcription from a 5 kb *Gata4* promoter. These results suggest that RERE might regulate the expression of GATA4 in the AV canal, at least in part, through its positive effects on *Gata4* transcription.

Although several lines of evidence suggested that retinoic acid might potentiate the effects of RERE on GATA4 transcription, we did not see an effect of retinoic acid treatment on the transcriptional activity from a 5 kb *Gata4* promoter in NIH3T3 cells. This suggests that RERE might be working through a different signaling pathway to promote transcription of *Gata4*.

### High VSD penetrance and reductions in GATA4 protein levels in the AV canal require decreased expression of RERE in cells other than the endocardium

We have shown that GATA4 protein levels are reduced in the AV canals of *Rere*^om/eyes3^ embryos at E10.5. This suggests that reductions in GATA4 expression might be contributing to the high penetrance of VSDs seen in *Rere*^om/eyes3^ embryos. In contrast, GATA4 levels were not found to be reduced in *Rere*^flox/flox^;Tie2-Cre embryos. Because *Rere*^flox/flox^;Tie2-Cre embryos have hypocellular endocardial cushions owing to decreased levels of EMT, and a subset go on to develop VSDs, these results suggest that RERE likely functions within endothelial cells to affect the transcription of genes other than *Gata4* that are required for EMT. Additional studies will be needed to identify these EMT-related gene targets.

These results also suggest that decreased expression of RERE in cells other than the endocardium plays a role in regulating the expression of GATA4 in the AV canal. This is not entirely unexpected, because RERE is expressed not only in the endocardium, but also in the myocardium and epicardium of the AV canal. Alterations affecting each of these cell layers can contribute to the development of VSDs, and crosstalk between tissue layers is required for proper formation of the AV endocardial cushions (Anderson et al., 2003; Combs and Yutzey, 2009). Hence, it is possible that decreased RERE expression in these cell layers leads, secondarily, to decreases in GATA4 expression in the AV canal.

GATA4 has also been shown to play a role in the development of the AV septum through its effects in the myocardium and second heart field (Misra et al., 2014; Zhou et al., 2017). Because RERE is also expressed in these tissues (Zoltewicz et al., 2004), it is possible that RERE contributes to the development of the AV septum by directly regulating *Gata4* transcription or secondarily affecting GATA4 expression in these tissues. Additional studies will be needed to clearly define the role of RERE in these and other cardiac-related tissues.

### Variability of RERE-associated VSDs

Septal defects are the most common forms of CHD seen in individuals with mutations affecting *RERE* (Fregeau et al., 2016; Jordan et al., 2018). These defects are not typically associated with abnormal valves or conotruncal defects. Similarly, VSDs are commonly seen in *Rere*^flox/flox^;Tie2-Cre and *Rere*^om/eyes3^ embryos at E15.5, and are not typically associated with valvular defects. However, additional studies will be needed to determine whether *Rere*^om/eyes3^ and *Rere*^flox/flox^;Tie2-Cre mice have defects in valve maturation.

The VSDs identified in *Rere*^flox/flox^;Tie2-Cre embryos are not associated with conotruncal defects. In contrast, conotruncal defects are commonly associated with the VSDs seen in *Rere*^om/eyes3^ and *Rere*^flox/flox^;*Nkx2-5*^Cre/+^ embryos. These VSDs might be generated, at least in part, through a different mechanism that might also contribute to the defect in cardiac looping seen in *Rere*^om/om^ embryos (Kim et al., 2013; Zoltewicz et al., 2004).

### Conclusions regarding the morphogenetic and molecular mechanisms by which RERE deficiency causes septal defects

Taken together, our results suggest that RERE functions in the AV canal to positively regulate the expression of GATA4, and that deficiency of RERE leads to the development of VSDs through its effects on EMT and mesenchymal cell proliferation. However, the cell-autonomous role of RERE in promoting EMT in the endocardium must be mediated by its effects on the expression of proteins other than GATA4.

## MATERIALS AND METHODS

### Mice

All experiments using mouse models were conducted in accordance with the recommendations in the Guide for the Care and Use of Laboratory Animals of the National Institutes of Health (NIH). The associated protocols were approved by the Institutional Animal Care and Use Committee of Baylor College of Medicine (Animal Welfare Assurance #A3832-01).

Wild-type C57BL/6 embryos were used to define the expression pattern of RERE and GATA4 in the heart. The generation of the *Rere om* null and *eyes3* alleles was described previously (Kim et al., 2013; Zoltewicz et al., 2004). Experiments using these alleles were conducted on a C57BL/6 background or mixed B6/SV129 background. Mice bearing the *Nkx2-5*-Cre knock-in allele, the transgenic Tie2-Cre allele, the Hprt-Cre allele and a *Gata4* null allele were described previously (Kisanuki et al., 2001; Molkentin et al., 1997; Moses et al., 2001; Tang et al., 2002).

The *Rere* flox allele was generated using recombineering (Liu et al., 2003). Briefly, a 9.6 kb fragment containing the second coding exon of *Rere* was retrieved into a pBluescript-derived plasmid containing 500 bp homology arms from BAC clone bMQ386d15 selected from the M37-129AB22 BAC library. A neomycin resistance cassette flanked by two loxP sites was then inserted 5′ to the second coding exon of *Rere*. Cre expression was induced, which resulted in recombination of the loxP sites leaving a single loxP site marked with an *Eco*R1 restriction site. A neomycin resistance cassette flanked by an frt site, and an frt and loxP site marked with a *Bgl*II restriction site, was then inserted 3′ to the second coding exon of *Rere* to generate the final targeting vector (Fig. S2). This targeting vector was linearized and electroporated into SV129/AB2.2 ES cells. Positive clones were identified by Southern blotting, injected into C57BL/6 blastocysts, and implanted into ICR outbred, pseudopregnant females. Highly chimeric males were mated to C57BL/6 females, and agouti pups were selected and genotyped for transmission of the *Rere* neo allele (Fig. S2). Mice bearing the *Rere* neo allele were crossed with mice bearing a *Rosa-FLPe* allele (Farley et al., 2000) for *in vivo* removal of the neo cassette and generation of the *Rere* flox allele. An *Rere* del allele was generated *in vivo* by crossing mice bearing the *Rere* flox allele to mice carrying an Hprt-Cre allele (Tang et al., 2002). In preparation for experiments on a C57BL/6 background, the B6/129S6 mice bearing the *Rere* flox allele were backcrossed for at least another six generations onto the C57BL/6 background.

The wild-type and *Rere* flox alleles were genotyped in PCR reactions using the following for primers: *Rere* true WT (5′-CGGGAAGAGTGTCTTTGGAGATGAC-3′), *Rere* del control (5′-GCAAAAGCCAATCACCACATCCTCTCC-3′), *Rere* 3′ common (5′-GTTCAGGGATTAGGCAGTATCTG-3′) and *Rere* flox (5′-CTTGCAGCTTCGAATTCCGAAGTTCC-3′), which generates a 335 bp wild-type product and a 144 bp *Rere* flox product. The wild-type and *Rere* del alleles were genotyped in PCR reactions using the following primers: *Rere* 5′ common (5′-TCACTGCACCCAGAGAGAGCTACAG-3′), *Rere* del control (5′-GCAAAAGCCAATCACCACATCCTCTCC-3′) and *Rere* 3′ common (5′-GTTCAGGGATTAGGCAGTATCTG-3′), which generates a 433 bp wild-type product and a 145 bp *Rere* del product.

### Preparation of paraffin-embedded tissue sections and tissue staining

Embryos were harvested and fixed with Buffered Formalde-Fresh Solution (Fisher Scientific, Pittsburgh, PA, USA) for 1 day at 4°C. After washing with phosphate-buffered saline solution (PBS), tissues were dehydrated in ethanol and embedded in paraffin. Paraffin-embedded tissue blocks were sectioned at 6 µm with an RM2155 microtome (Fisher Scientific). Hematoxylin and Eosin staining was used for histological analyses.

### Histological analyses

Sagittal sections stained with H&E staining were used for quantification of mesenchymal cell number. Mesenchymal cells in the AV endocardial cushions were counted and normalized to the area of the AV cushion on each section using ImageJ software (NIH; https://imagej.nih.gov/ij/).

For immunohistochemical analyses, tissue sections were deparaffinized and blocked with 1× PBS containing 1% bovine serum albumin (BSA) and 5% normal donkey serum for 1 h at room temperature. Sections were then incubated with anti-RERE (sc-98415, 1:200; Santa Cruz Biotechnology, Santa Cruz, CA, USA), anti-phospho-histone H3 (pHH3) (#9701, 1:200; Cell Signaling Technology, Danvers, MA, USA), anti-cleaved caspase-3 (#9664, 1:200; Cell Signaling Technology), or anti-GATA4 (sc-25310, 1:200; Santa Cruz Biotechnology) antibodies diluted in the same blocking solution (1% BSA and 5% normal donkey serum in 1× PBS) overnight at 4°C. After washing with 1× PBS, the sections were incubated with biotin-conjugated anti-rabbit IgG or biotin-conjugated anti-mouse IgG (Jackson ImmunoResearch, West Grove, PA, USA).

Immunoreactivity of each antibody was visualized using a tyramide signal amplification (TSA) kit (Invitrogen, Grand Island, NY, USA) containing Alexa Fluor 488 dye or Alexa Fluor 568 dye for fluorescent labeling according to the manufacturer's instructions. Images were acquired on a Zeiss Axioplan microscope equipped with an AxioCam digital camera and imaging system (Carl Zeiss Microscopy, Gena, Germany). For RERE expression studies, three slides, each containing at least three sections, were analyzed from each of three E9.5 embryos, five E10.5 embryos, three E15.5 embryos and three E15.5 embryos.

To quantify the proliferation and apoptosis of mesenchymal cells, mesenchymal cells were labeled with anti-pHH3 or anti-cleaved caspase-3 antibodies, respectively. Positive cells were counted and normalized to the total number of cells in the AV endocardial cushions using ImageJ. For statistical analysis, unpaired two-tailed Student's *t*-test was used. The standard deviation of the mean (±s.d.) was plotted as an error bar in the associated graphs.

Additional information on the antibodies used in this study can found in Table S4.

### AV canal explant culture assays

The AV canal explants were cultured on collagen gels for analysis of EMT essentially as previously described (Xiong et al., 2012). Briefly, collagen gels were made from a solution of type I rat tail collagen (Corning, Corning, NY, USA) in 12-well Costar tissue culture plates (Corning). Collagen gels were soaked with culture medium containing OptiMEM-1 (Thermo Fisher Scientific, Waltham, MA, USA), 1% FBS (Thermo Fisher Scientific), 100 U/ml penicillin (Thermo Fisher Scientific) and 100 µg/ml streptomycin (Thermo Fisher Scientific) overnight at 37°C. The culture medium was removed from the gels prior to the dissection of the AV canals. AV canals were dissected from E9.5 embryos, transferred to the collagen gels, and incubated for 48 h in 5% CO_2_ at 37°C. At 24 h and 48 h of culture, images were acquired using an Axiovert 40 CFL inverted microscope equipped with an AxioCam digital camera and imaging system (Carl Zeiss Microscopy). Elongated or spindle-shaped mesenchymal cells located outside of the endocardial sheath were counted as migrating mesenchymal cells. For statistical analysis, unpaired two-tailed Student's *t*-test was used (error bars in associated graphs show the s.d.).

### Gene expression analysis

RT-qPCR analyses were performed as previously described (Beck et al., 2013; Scott et al., 2007). Briefly, total RNA was extracted from E10.5 embryonic hearts using an RNeasy Micro Kit (Qiagen, Germantown, MD, USA) according to the manufacturer's recommendations. Complementary DNA (cDNA) was synthesized from each RNA sample by using amfiRivert cDNA synthesis Platinum Master Mix (GenDEPOT, Katy, TX, USA). qPCR analyses were performed using LightCycler FastStart DNA Master SYBR Green I Mix (Roche Diagnostics, Mannheim, Germany) on a LightCycler 1.5 (Roche Diagnostics, West Sussex, UK). The following primers were used for amplification: forward, 5′-CCAGCGGTAACTCCAGCAA-3′, reverse, 5′-GGACAGCTTCAGAGCAGACA-3′ for *Gata4*; forward, 5′-TGTTTAGGCCAAGCAGAGGT-3′, reverse, 5′-CGCTCCAAGTAGCGTCTCAT-3′ for *Erbb3*; forward, 5′-GCACCCTCCCTAACCCTCTA-3′, reverse, 5′-ACTGCTGCTCCATGGCTAGT-3′ for *Rere*; forward, 5′-GGAGGCGAAGAAAAGCAACAG-3′, reverse, 5′-CAACACTAGAAGACAGCGGGTC-3′ for *Bmp2*; forward, 5′-AGGAGGAGGAGGAAGAGCAG-3′, reverse, 5′-TGCTGCTGAGGTTGAAGAGG-3′ for *Bmp4*; forward, 5′-ACCTCTTGCCAGCCTACATG-3′, reverse, 5′-TGCTGCTGTCACTGCTTCTC-3′ for *Bmp5*; forward 5′-TCAGTGGCCCTAATTGCCAG-3′, reverse, 5′-ACCTCGCAGGTTTGACCTTG-3′ for *Notch1*; forward, 5′-AGATGCACATTCGAACCCAC-3′, reverse, 5′-GTCTGCAGATGAGCCCTCAG-3′ for *Snai2*; forward, 5′-GAGGTGTGTGAGGACAGTGG-3′, reverse, 5′-AACTCCTCACAGCTTCTGCC-3′ for *Nfatc1*; forward, 5′-TCTGCACGACCTGTGGAAAG-3′, reverse, 5′-AGCCAGACTCTTGGTGCTTG-3′ for *Snai1*; forward, 5′-GCATTGAGGCTGTGCGGAAGG-3′, reverse 5′-CATGTGCTGCTGCTTCCATAAG-3′ for *Polr2a* (RNA polymerase II), which served as a reference gene. The 2^−ΔΔ*CT*^ method was used for RT-qPCR analysis. For statistical analysis, unpaired two-tailed Student's *t*-test was used (error bars in associated graphs show the s.d.). A Benjamini–Hochberg procedure with a 10% false discovery rate was performed as a means of accounting for multiple comparisons using a publically available spreadsheet available online at http://www.biostathandbook.com/multiplecomparisons.html (accessed on 18 June, 2018).

### Plasmids and siRNA

The *RERE* expression vector used in this study was kindly provided by Dr Chih-Cheng Tsai (University of Medicine and Dentistry of New Jersey-Robert Wood Johnson Medical School, Piscataway, NJ, USA). The luciferase reporter vector containing the 5 kb *Gata4* promoter region was a gift from Dr Robert S. Viger (Laval University, Quebec City, Quebec, Canada). A pRL-TK-based Renilla luciferase vector was purchased from Promega (Madison, WI, USA). The mammalian expression vector pCDNA3.1 was obtained from Thermo Fisher Scientific. *Rere* siRNA (SMART Pool) and nontargeting siRNA pools were purchased from Dharmacon (Lafayette, CO, USA).

### Western blot analyses

HEK293T cells or NIH3T3 cells were homogenized with lysis buffer containing 20 mM Tris-HCl (pH7.5), 150 mM NaCl, 1 mM Na_2_EDTA, 1% Triton X-100, 2.5 mM sodium pyrophosphate, 1 mM Na_2_VO_4_ and Complete Protease Inhibitor Cocktail (Roche Applied Bioscience, Mannheim, Germany) as per the manufacturer's instructions. Protein extracts prepared from homogenates were resolved by sodium dodecyl sulfate–polyacrylamide gel electrophoresis and transferred to nitrocellulose membranes. These membranes were probed with the following antibodies: anti-RERE (sc-98415, 1:500; Santa Cruz Biotechnology) and anti-GAPDH (#2810, 1:2000; Cell Signaling Technology). Each blot was visualized using a SuperSignal West Pico Chemiluminescent detection kit (Thermo Fisher Scientific) according to the manufacturer's instructions.

### Cell culture and reporter gene assay

HEK293T cells used in this study were kindly provided by Dr Brendan Lee (Baylor College of Medicine, Houston, TX, USA). These cells were cultured in Dulbeco's modified Eagle's medium (DMEM; Thermo Fisher Scientific), 10% FBS, 100 U/ml penicillin and 100 µg/ml streptomycin. NIH3T3 cells used in this study were kindly provided by Dr Suzanne A. W. Fuqua (Baylor College of Medicine). These cells were cultured in DMEM, 10% calf serum (Thermo Fisher Scientific), 100 U/ml penicillin and 100 µg/ml streptomycin. All cells were routinely subjected to mycoplasma testing using MycoAlert™ Mycoplasma Detection Kit (Lonza, Allendale, NJ, USA).

For transient transfection of plasmids, Lipofectamine^TM^ 3000 reagent (Thermo Fisher Scientific) was used according to the manufacturer's recommendations. In brief, the diluted Lipofectamine^TM^ 3000 reagent was mixed with diluted DNA solutions containing plasmids and P3000^TM^ reagent. Plasmids-lipofectamine complex was added to HEK293T cells and allowed to incubate for 30 h. For transient transfection of siRNA, Lipofectamine^TM^ 3000 reagent was diluted and mixed with siRNA/DNA solutions or siRNA solutions. DNA/siRNA-lipofectamine complex or siRNA-lipofectamine complex was applied to NIH3T3 cells and allowed to incubate for 48 h.

To measure luciferase activity from transfected HEK293T cells or NIH3T3 cells, we used the Dual-Luciferase Reporter Assay System (Promega), which allowed us to normalize for transfection efficiency based on Renilla luciferase levels. Cell extracts were prepared according to the manufacturer's recommendations. Luciferase activity was quantified using a FLUOstar OPTIMA fluorometer (BMG LABTECH, Ortenberg, Germany). To determine the effects of retinoic acid on luciferase activity, NIH3T3 cells were treated with 0.25, 0.5, 1.0, 2.0 and 3.0 µM of all trans-retinoic acid (Sigma-Aldrich, St. Louis, MO, USA) for 30 h starting at 24 h post-transfection. All experiments were performed in triplicate. For statistical analysis, unpaired two-tailed Student's *t*-test was used (error bars in associated graphs show the s.d.).

## Supplementary Material

Supplementary information

First Person interview
