# Supplementary material for: RERE deficiency leads to decreased expression of GATA4 and the development of ventricular septal defects
Source: Dis Model Mech. 2018 Aug 28;11(9):dmm031534. doi: 10.1242/dmm.031534 (PMC6176990; doi:10.1242/dmm.031534)
Supplement: Supplementary information [file dmm-11-031534-s1.pdf]

FIRST PERSON

First person – Bum Jun Kim

First Person is a series of interviews with the first authors of a selection of papers published in Disease Models & Mechanisms, helping early-career researchers promote themselves alongside their papers. Bum Jun Kim is first author on 'RERE deficiency leads to decreased expression of GATA4 and the development of ventricular septal defects', published in DMM. Bum Jun is a staff scientist in the lab of Daryl A. Scott at Baylor College of Medicine, Houston, TX, USA, investigating the pathogenic role of RERE in phenotypes caused by 1p36 deletions, particularly in congenital heart defects, brain abnormalities and eye defects.

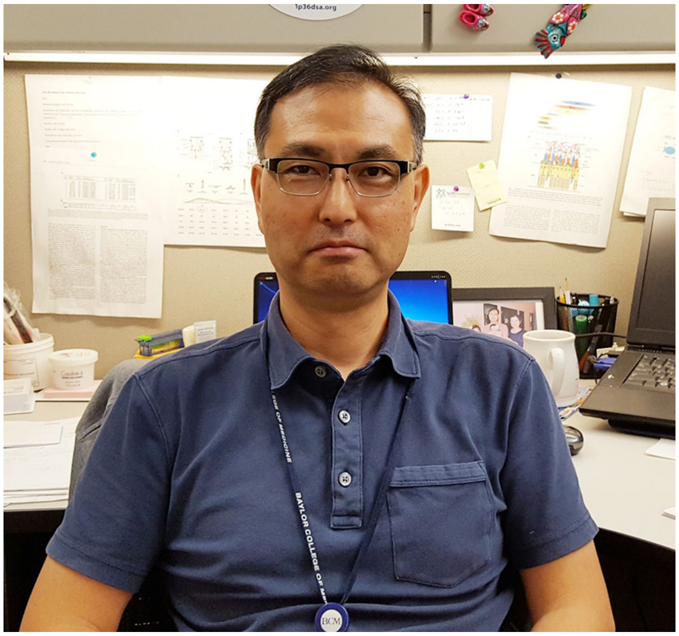

Bum Jun Kim

How would you explain the main findings of your paper to non-scientific family and friends?

Chromosome 1p36 deletion syndrome is a genetic disorder found in children with an incidence of 1 in 5000 newborns, and results in various problems in the heart, the brain, growth, vision and intelligence. We have discovered that a gene, named *RERE*, is one of the causative genes for the symptoms seen in individuals with this syndrome, through use of a mouse model in which function of RERE is limited. During heart development, the heart is divided into the right chamber and left chamber by an internal divider called the ventricular septum. The main finding of this study is that the two chambers are incompletely divided by abnormal development of the ventricular septum in RERE-deficient mice. We also provide evidence to show that *RERE* regulates production of a protein called GATA4. Mutations in the *GATA4* gene are already known to be associated with ventricular septum abnormalities in humans. We learned that in our mouse models, *RERE* controls development of the ventricular septum by modulation of GATA4 protein production.

What are the potential implications of these results for your field of research?

Although several lines of evidence support that RERE clearly plays a crucial role in septal development, the morphogenetic and molecular mechanisms by which RERE deficiency causes septal defects are not known. This study shows that RERE functions to positively regulate the expression of GATA4 in the developing atrioventricular canal and that a deficiency of RERE leads to the development of ventricular septal defects (VSDs) through its effects on epithelia-to-mesenchymal transition and mesenchymal cell proliferation. Our findings imply that RERE-deficient mice are not only useful for investigating the role of RERE in the development of VSDs, but also to understand the molecular mechanisms that cause other phenotypes associated with 1p36 deletions.

What are the main advantages and drawbacks of the model system you have used as it relates to the disease you are investigating?

RERE-deficient mice mimic various defects seen in individuals with 1p36 deletions, which means this mouse model is useful to provide insights into how RERE deficiency causes these phenotypes. We also generated a tissue-specific knockout mouse model using a *Rere* flox allele and the Cre allele, then demonstrated the usefulness of this model to find the precise mechanisms that are affected by RERE deficiency in a tissue-specific manner.

"Understanding of tissue-specific molecular mechanisms or pathways is a fundamental step to finding the molecules or players engaged in the pathogenesis of diseases."

What has surprised you the most while conducting your research?

VSDs are consistently identified in RERE-deficient mice, but the level of VSD penetrance varied when different Cre mice were used to delete *Rere* from specific regions of the heart. Ablation of *Rere* from the myocardium of the heart resulted in VSDs with 100% penetrance but VSDs were only identified in a few mice where *Rere* was depleted in the endocardium of the heart. These data suggest that RERE may regulate different pathways or targets based on its location, even in the same organ. Understanding of tissue-specific molecular mechanisms or pathways is a fundamental step to finding the molecules or players engaged in the pathogenesis of diseases.

Describe what you think is the most significant challenge impacting your research at this time and how will this be addressed over the next 10 years?

The availability of genetic information from individuals with 1p36 deletion for clinical studies or research is robustly increasing with advances in genetic analysis tools such as next-generation sequencing

Bum Jun Kim's contact details: Department of Molecular and Human Genetics, Baylor College of Medicine, Houston, TX 77030, USA.
E-mail: bumjunk@bcm.edu

(NGS). Even though it is likely that routine research will identify variants in the *RERE* gene or further genes associated with 1p36 deletion phenotypes, gaining an understanding of each variant's functional effect or molecular mechanisms is still a limiting factor to the application of this genetic information for diagnosis, prognosis or development of therapeutic approaches, including gene therapy. Prediction models based on big data may provide an answer as to the physiological meaning of the variants identified in patients.

What's next for you?

Using RNA sequencing data obtained from *Rere* mutant mouse models, our next challenge is to build a map showing the genetic networks between genes that are required for development of the congenital heart defects seen in individuals with 1p36 deletion. Eventually, we hope to obtain molecular evidence that will enable us to predict genotypes and phenotypes.

Reference

Kim, B. J., Zaveri, H. P., Jordan, V. K., Hernandez-Garcia, A., Jacob, D. J., Zamora, D. L., Yu, W., Schwartz, R. J. and Scott, D. A. (2018). RERE deficiency leads to decreased expression of GATA4 and the development of ventricular septal defects. *Dis. Model. Mech.* **11**: dmm031534.
